# Redo mitral valve replacement through minithoracotomy on ventricular fibrillation: Bailout for a nightmare Redo

**DOI:** 10.1002/ccr3.3384

**Published:** 2020-12-16

**Authors:** João Pedro Monteiro, Sara Simões Costa, Nelson Santos Paulo, Rodolfo Pereira

**Affiliations:** ^1^ Cardiothoracic Surgery Centro Hospitalar de Vila Nova de Gaia/Espinho Vila Nova de Gaia Portugal

**Keywords:** mitral valve procedures, redo, thoracotomy, ventricular fibrillation

## Abstract

A 56‐year‐old woman entered the emergency department due to worsening dyspnea. Severe mitral regurgitation and pulmonary artery dilation with flow compatible with fistula were observed by transthoracic and transesophageal echocardiography. The patient had history of an ALCAPA (anomalous left coronary artery from pulmonary artery) syndrome having undergone coronary artery bypass grafting (saphenous venous graft to left anterior descending artery) 30 years before. Coronary angiography and computed tomography revealed patency of the graft, with the dilated vein running across the front of the ascending aorta and being responsible for the perfusion of the left anterior descending artery and circumflex artery. We resent this case for discussion of which surgical strategy/options are available in order to treat the mitral valve and avoid injuring the patent graft.

## Graphical abstract

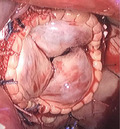

## 1 CASE REPORT

A 61‐year‐old woman entered the emergency department due to worsening dyspnea. Severe mitral regurgitation was observed by transesophageal echocardiography. The patient had history of an ALCAPA (Anomalous Left Coronary Artery from Pulmonary Artery) syndrome having undergone coronary artery bypass grafting (saphenous venous graft to left anterior descending artery) 30 years before. Coronary angiography and computed tomography revealed patency of the graft, with the dilated vein running across the front of the ascending aorta and being responsible for the perfusion of the left anterior descending and circumflex arteries. To avoid injuring the patent graft, mitral valve replacement under ventricular fibrillation without aortic cross‐clamping was performed through a right minithoracotomy approach. Postoperative course was uneventful, and she was discharged on hospital day 7. This method appears safe, effective, and useful for avoiding secondary injuries in patients with severe mitral regurgitation, previous sternotomy, and patent bypass grafts.

**FIGURE 1:**
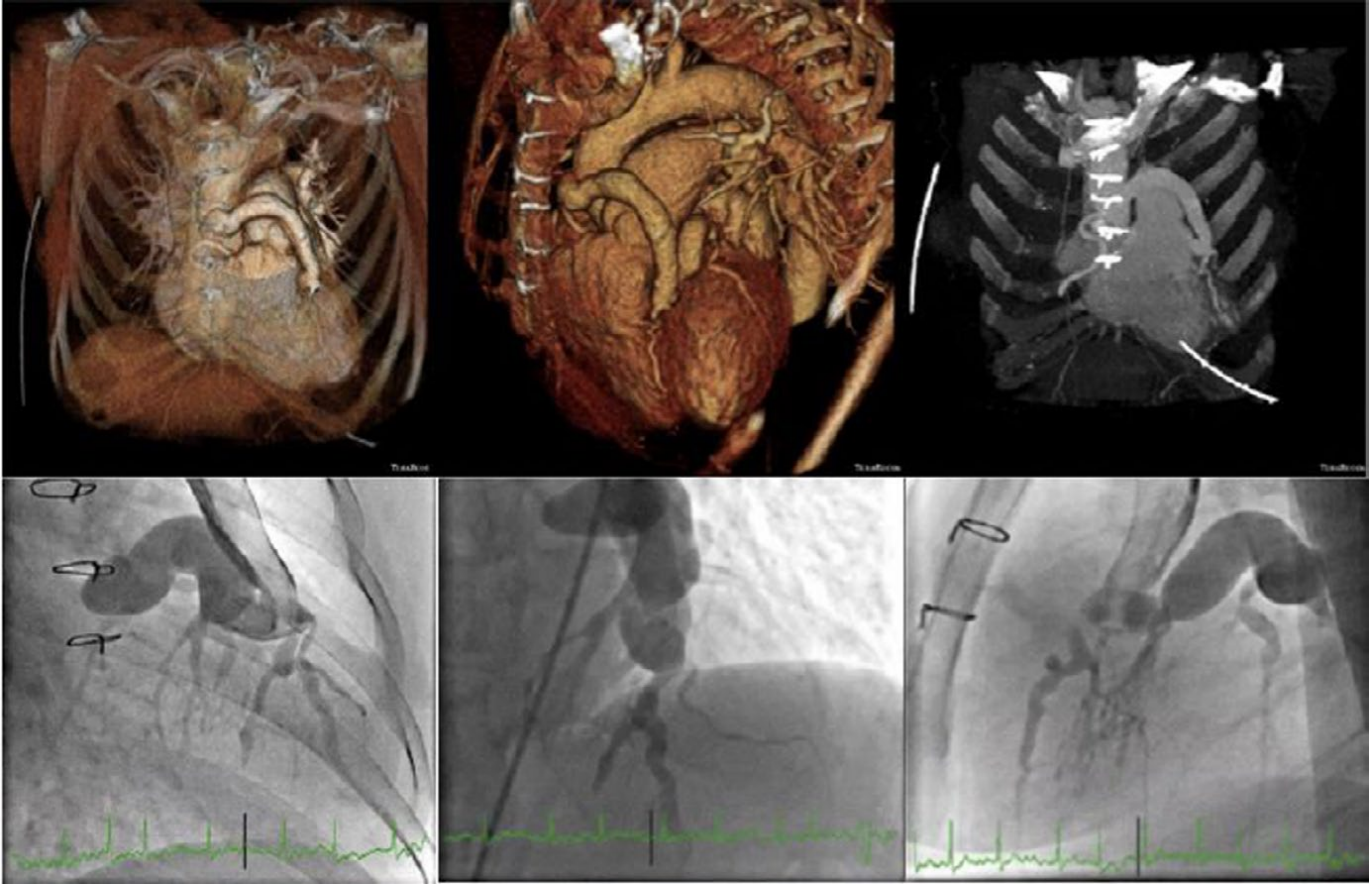
Computed tomography and coronary angiography showing the dilated patent coronary graft (saphenous vein to left anterior descending artery)

We report a case of a 61‐year‐old woman with worsening dyspnea and history of an ALCAPA (Anomalous Left Coronary Artery from Pulmonary Artery) syndrome having undergone coronary artery bypass grafting (saphenous venous graft to left anterior descending artery) 30 years before. Transesophageal echocardiography revealed severe mitral regurgitation. Coronary angiography and computed tomography showed patency of the graft, with the dilated vein running across the front of the ascending aorta and being responsible for the perfusion of the left anterior descending and circumflex arteries. (Figure 1). In order to avoid injuring the graft, we performed the mitral valve replacement (MVR) through a right minithoracotomy approach under ventricular fibrillation (VF) without aortic cross‐clamping.

## 2 SURGICAL TECHNIQUE

**FIGURE 2:**
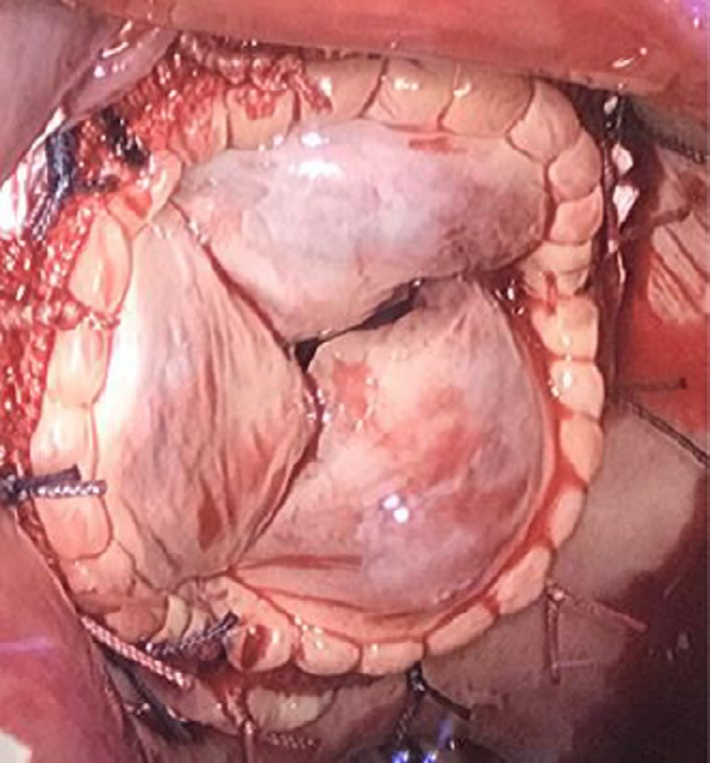
Surgeon's view of the implanted prosthetic biologic St Jude^®^ #33 valve

A double lumen endotracheal tube was used for intubation and transesophageal echocardiography was placed for cardiac monitoring. The chest was opened through a right minithoracotomy (skin incision ≤7 cm) under one lung ventilation at the 4th anterolateral intercostal space. The endoscopic port was placed at the right 3rd anterior intercostal space and used as a CO2 port. Cardiopulmonary bypass was instituted through peripheral cannulation (right femoral artery and vein) using vacuum‐assisted venous drainage. After cooling to 22°C in order to induce VF, a left atriotomy between the phrenic nerve and pulmonary veins was performed. MVR was performed in standard fashion using instruments for minimally invasive mitral surgery. After a prosthetic biologic St Jude^®^ #33 valve was sewn into place (Figure 2), the left atriotomy was closed with slow filling and de‐airing. When rewarming was completed, cardioversion was performed. After recovery to sinus rhythm, cardiopulmonary bypass was terminated and the femoral cannulas removed. A right pleural chest tube was positioned, and the incision was closed.

No intraoperative complications were registered. The patient had good in‐hospital progression and discharge on postoperative day 7.

## 3 DISCUSSION

Conventional reoperative MVR by median sternotomy has several challenges as it requires dissection to the apex, aortic clamp, and myocardial protection.^1^, ^2^ In the presence of adhesions, this approach carries increased risk of injury of major cardiac structures (right ventricle, innominate vein, and bypassed grafts).^1^, ^2^ This case revealed a very dilated vein running close to the sternum and across the front of the ascending aorta. (Figure 1) These anatomical proximities and the fact that this graft is responsible for the perfusion of all left coronary territory are high predictive factors of surgical risk and mortality in a re‐sternotomy.

Minimally invasive mitral surgery is associated with a mortality rate similar to that for sternotomy but reduced length of intensive care unit and hospital stays, fewer blood transfusions, earlier recovery of daily activities, and improved quality of life.^1^, ^2^ The right minithoracotomy approach can achieve an excellent operative view of the mitral valve without requiring dissection of adhesions and has demonstrated to be safe and with similar results to re‐sternotomy.^1^, ^2^ By performing the procedure under VF with systemic hypothermia, aortic cross‐clamping and interruption of the graft flow are unnecessary, not necessarily compromising myocardial protection, thus, avoiding dangerous dissection around the aorta and the dilated patent graft.^1^, ^2^ Even though myocardial protection can still be a concern to surgeons, the safety of MVR under VF has been documented in these particular cases of functioning grafts.^3^ Excluding cases of aortic insufficiency, which can make this technique unfeasible because of retrograde flow obstructing the operative field, when comparing to MVR with cardioplegic arrest, the efficiency and safety of this method are also reported.^1^, ^2^, ^3^

Current knowledge and the success of our case suggest that reoperative MVR under VF without aorta cross‐clamping through a right minithoracotomy is a safe, reproducible, and effective option for patients requiring redo mitral valve surgery, especially when presenting anatomical characteristics that increase the risk of re‐sternotomy such as coronary bypass grafts.

## CONFLICT OF INTEREST

None declared.

## ETHICAL APPROVAL

All ethical concerns were respected throughout the ellaboration of this case report.
